# Label Free Detection of Sensitive Mid-Infrared Biomarkers of Glomerulonephritis in Urine Using Fourier Transform Infrared Spectroscopy

**DOI:** 10.1038/s41598-017-04774-7

**Published:** 2017-07-04

**Authors:** Mei-Ching Yu, Peter Rich, Liberty Foreman, Jennifer Smith, Mei-Shiuan Yu, Anisha Tanna, Vinod Dibbur, Robert Unwin, Frederick W. K. Tam

**Affiliations:** 1 Division of Pediatric Nephrology, Department of Pediatrics, Chang Gung Memorial Hospital at Lin-Kou and Chang Gung University, Taoyuan, Taiwan; 2 Imperial College Kidney and Transplant Centre, Renal and Vascular Inflammation Section, Department of Medicine, Imperial College London, London, United Kingdom; 3 Institute of Structural and Molecular Biology, Faculty of Life Science, University College London, London, United Kingdom; 4 Department of Microbiology, School of Medicine, Tzu-Chi University, Hualien, Taiwan; 5 UCL Center for Nephrology, University College London Medical School, Royal Free Campus and Hospital, London, United Kingdom

## Abstract

More reliable biomarkers using near-patient technologies are needed to improve early diagnosis and intervention for patients with renal disease. Infrared (IR) vibrational spectroscopy/microspectroscopy is an established analytical method that was first used in biomedical research over 20 years ago. With the advances in instrumentation, computational and mathematical techniques, this technology has now been applied to a variety of diseases; however, applications in nephrology are just beginning to emerge. In the present study, we used attenuated total reflectance Fourier transform infrared (ATR-FTIR) spectroscopy to analyze urine samples collected from rodent models of inflammatory glomerulonephritis (GN) as well as from patients with crescentic GN, with the aim of identifying potential renal biomarkers; several characteristic mid-IR spectral markers were identified in urine samples. Specifically, a 1545 cm^−1^ band increased in intensity with the progression and severity of GN in rats, mice and humans. Furthermore, its intensity declined significantly in response to corticosteroid treatment in nephritic rats. In conclusion, our results suggest that specific urinary FTIR biomarkers may provide a rapid, sensitive and novel non-invasive means of diagnosing inflammatory forms of GN, and for real-time monitoring of progress, and response to treatment.

## Introduction

Despite ongoing efforts to develop effective treatments for kidney diseases, the number of patients with chronic kidney disease (CKD) and end-stage renal failure (ESRF) continues to grow worldwide^1^. The conventional renal biomarkers of serum creatinine (Scr) and proteinuria (or albuminuria) are used widely, but are relatively insensitive in that they change later in disease. Scr is also affected by several non-renal factors such as age, gender and body mass^2^. For many patients, by the time a rise in Scr is detected, 50% of renal function has been lost and the presence and extent of proteinuria is highly variable^3^; moreover, although proteinuria can correlate with disease severity, it cannot distinguish between acute and chronic forms of injury^4, 5^. The result is that renal disease is often detected too late for any beneficial therapeutic intervention, which can lead to poor renal outcomes. Renal biopsy is currently a reliable method of diagnosing the nature and extent of renal disease, but repeated biopsy is limited in a clinical setting, because of its invasiveness. Thus, sensitive non-invasive biomarkers of early renal injury are needed for prompt detection and monitoring of patients, and for better clinical trial design.

Fourier transform infrared (FTIR) spectroscopy measures absorption of IR light caused by transitions between energy levels of molecular vibrational normal modes^6, 7^. FTIR spectroscopy covers the 6000–200 cm^−1^ region of the electromagnetic spectrum. Particularly useful when analyzing biological samples is the mid-IR fingerprint region of 1800-900 cm^−1^ where many molecules exhibit characteristic absorption bands. For example, protein peptide bonds have amide I (1690-1600 cm^−1^) and amide II (1575-1480 cm^−1^) bands; deoxyribonucleic acid has symmetric and asymmetric PO_2_^−^ stretches at 1244 cm^−1^ and 1089 cm^−1^, respectively^7–9^. FTIR spectroscopy and FTIR microscopy imaging have been increasingly applied to diseases such as cancer by analyzing biofluids, tissue sections or cells^10–19^. The spectral data can provide rapid quantitative and qualitative measurements of biochemical changes caused by pathologies and without chemical modification of the original fluid or tissue samples. FTIR spectroscopy/microspectroscopy has been investigated recently as a potential diagnostic tool in various forms of kidney diseases^20–23^. Its main advantages are that it is ‘manipulation-free’, chemically non-destructive, cost-effective, and potentially operator-independent, unlike many other spectroscopy-based analytical platforms applied to urine, such as mass spectroscopy and nuclear magnetic resonance. However, despite a continuous increase in applications of biomedical spectroscopy, several challenges encountered during clinical-translational research should be considered to overcome them. For instance, large-scale randomised control trials and incorporation with current standard diagnositc methods are required to prevent the bias from small sample studies, and further validate spectral biomakers or signatures for diagnositc or prognostic purposes^7, 24^. Besides, appropriate standarization (e.g. sample spectral collection and preanalytic processing), the roubustness of analytic methods, and automation will be helpful to facilitate real clinical translation with the respect to technical issue^7, 25, 26^.

In the present study, our aim was to identify specific FTIR spectral markers of renal injury in rodent models of acute and progressive glomerulonephritis (GN), and human inflammatory (crescentic) GN. Promising markers were assessed as a means of monitoring therapeutic responses and their relationship to extents of proteinuria as measured by standard clinical methods.

## Methods

### Animal models of glomerulonephritis (GN)

All animal methods were carried out in accordance with the relevant guidelines of Animals (Scientific Procedures) Act 1986 authorized by the Home Office, United Kingdom (UK). The animal experimental protocols were approved by Imperial College London, UK.

### The rat model of progressive crescentic GN

Wistar Kyoto (WKY) rats (Charles River Laboratories, London) weighing around 200–300 gm were used. Nephrotoxic nephritis (NTN) was induced in male WKY rats by a single intravenous injection of 0.1 ml rabbit anti-rat glomerular basement membrane antiserum. NTN in WKY rats is a reproducible rodent model of progressive glomerulonephritis, presenting histological features similar to human crescentic GN^27^. Following the injection of nephrotoxic serum (NTS), the rats were divided into four groups for study at different time points after injection that have distinct pathophysiological characteristics: (1) NTN at 8 days (d8 NTN, n = 20): acute inflammation, (2) NTN at 14 days (d14 NTN, n = 18): severe inflammation, (3) NTN at 21 days (d21 NTN, n = 17): early renal fibrosis, and (4) NTN at 28 days (d28 NTN, n = 18): late fibrosis and renal failure. Findings were compared with that from age-matched healthy normal WKY rats (n = 34). The rats were housed in metabolic cages with free access to food and water for 24 hours to collect urine. All of the urine samples were centrifuged at 1800 rpm (4 °C) for 5 minutes and then frozen at −20 °C for later measurements.

### A mouse model of spontaneous lupus nephritis

Female F1 hybrids of New Zealand Black and New Zealand White, (NZB x NZW) F1, mice were purchased from Harlan Laboratories UK. This mouse strain is capable of developing spontaneous autoimmune glomerulonephritis that resembles human lupus nephritis (LN)^28^. Twenty-four hour urine was collected from 14 (NZB x NZW) F1 mice aged between five and eight months. Urine dipstick testing in 11 mice ranged from negative to 1 + protein (i.e. 5 mice were negative, 5 mice had trace, and one mouse had 1 + protein, respectively); 2 mice had 2 + protein and 5 mice had 3 + to 4 + protein.

### Corticosteroid treatment in a rat model of experimental crescentic GN

Rats were grouped into: (1) normal WKY rats (n = 4) as controls; (2) NTN-treated WKY rats (n = 6) given dexamethasone treatment (0.25 mg/kg) by intraperitoneal (*ip*) injection (Sigma-Aldrich, UK) every 4 days; (3) NTN-treated WKY rats (n = 6) given vehicle only (0.25 mg/kg *ip* of sterile 1xPBS) every 4 days. Twenty-four hour urine was collected from nephritic rats following the injection of NTS at 4 days, 8 days, 14 days, 21 days and 28 days, and compared with normal and untreated rats at each time point.

### Clinical crescentic GN and other kidney diseases

Twenty-four antineutrophil cytoplasmic antibody (ANCA) vasculitis patients (26–82 years old) and 11 healthy volunteers (23–56 years old) were studied following written informed consent; this study protocol had been approved by the local research ethics committee (NRES Committee London-West London [REC reference 04/Q0406/25]) and the study was approved by Imperial College Healthcare NHS Trust. All methods were performed in accordance with the guidelines and regulations issued by the Joint Health Compliance Office, Academic Health Science Centre, Imperial College London and Imperial College Healthcare NHS Trust, United Kingdom. Urine samples were collected in outpatient clinics at the Hammersmith Hospital, Imperial College London, and centrifuged at 1800 rpm (4 °C) for 10 minutes. FTIR analysis was then done soon after collection. Healthy volunteers were not known to have kidney disease or hypertension or diabetes, and their dipstick urine tests were negative for protein, blood and sugar. The 24 vasculitis patients were biopsy proven crescentic GN showing various degrees of kidney damage and were being treated with corticosteroid, cyclophosphamide, azathioprine or mycophenolate mofetil, according to a standard protocol. The following information was retrieved from medical records: patient age, gender, urinalysis, urinary protein-to-creatinine ratio (uPCR), estimated glomerular filtration rate (eGFR), the titer of ANCA and results of histological study. Crescentic GN patients with eGFR ≧ 60 ml/min/1.73 m^2^ were considered to have mild renal impairment, whereas crescentic GN patients with eGFR <60 ml/min/1.73 m^2^ were considered to have moderate to severe renal impairment. Furthermore, the remission of ANCA-associated crescentic GN was defined by the absence of ANCA and urine dipstick-negative for protein or uPCR ≦ 45 mg/mmol. In contrast, active crescentic GN patients had positive ANCA and an uPCR > 45 mg/mmol.

### Proteinuria measurement

The sulphosalicylic acid (SSA) assay was used to quantify protein excretion in rat urine; for mouse urine, the level of proteinuria was determined using dipstick testing only.

### Urine Protein Ultrafiltration

Urine samples of normal, day 8 NTN and day 21 NTN rats were filtered through different sizes of AMICON Ultra 4 ml Centrifugal Filters (Merck Millipore, UK) with molecular weight cut-offs (MWCO) of 10,000 (10 K), 30,000 (30 K), 50,000 (50 K) and 100,000 (100 K), respectively. Subsequently, the ultrafiltrates were collected and then analyzed using ATR-FTIR.

### ATR-FTIR measurement

After thawing at room temperature, urine samples from rats, mice and humans were prepared by diluting with distilled water to 1:30, 1:20 or 1:40 (urine: water). Pipetting 5 µl of diluted urine was pipetted onto a silicon ATR prism (SensIR, 3 mm diameter, 3 reflections) and dried by nitrogen gas at a flow rate of 400 mL/min. Spectra were subsequently recorded with a Bruker Optics, Germany, IFS 66/S FTIR spectrometer equipped with an Mercury cadmium telluride (MCT)-A detector cooled using liquid nitrogen. All absorbance spectra were recorded in the 4000–800 cm^−1^ frequency range *versus* a clean prism background. Each spectrum was computed from the average of 500 interferograms at 4 cm^−1^ resolution using the clean prism surface for recording of the background spectrum. The prism surface was cleaned using distilled water and ethanol between samples. The resultant FTIR absorbance spectra were analyzed with Bruker 6.5 and Origin Pro 9.1 software.

### Urine spectral assignment: processing and analysis

**Figure 1:**
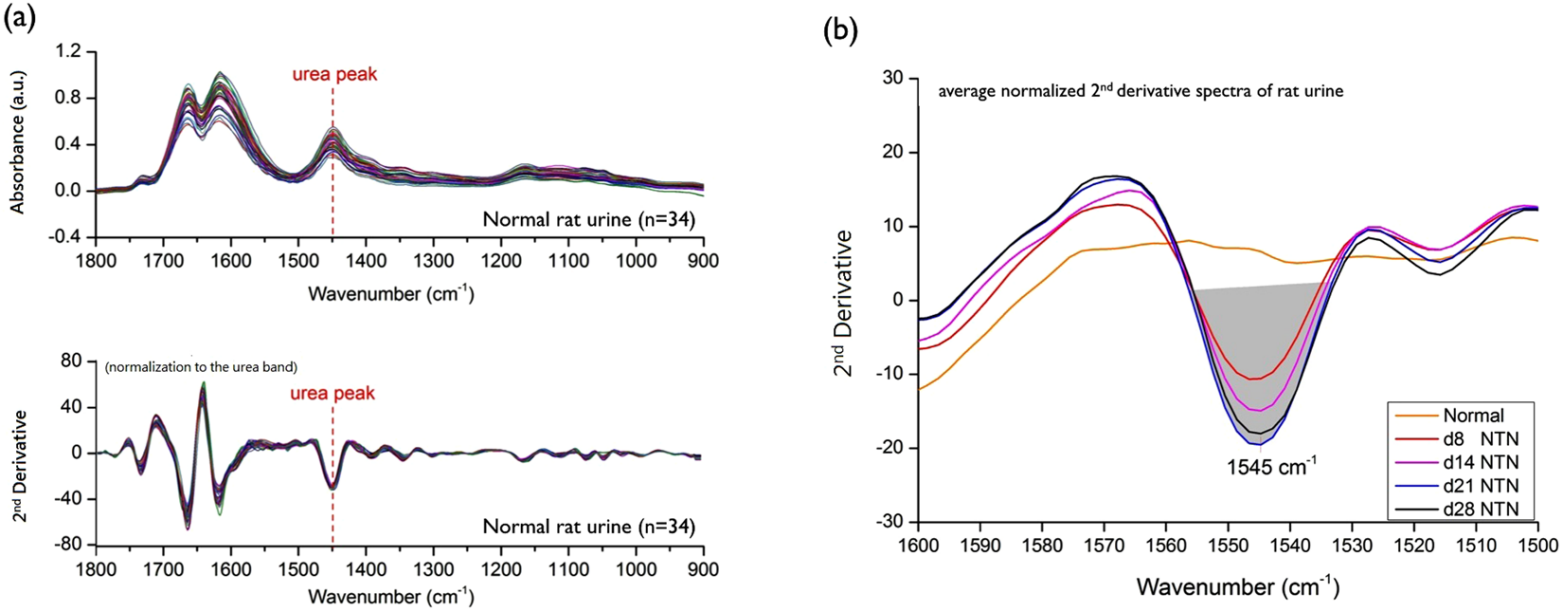
Fourier transform infrared (FTIR) analysis of rat urine: urine spectral normalization, second derivative conversion and integration. (**a**) Absorbance spectra of each dried urine sample were recorded from 34 normal rats (upper graph). Later, the urine spectra were individually normalized to the urea band at 1450 cm^−1^ (1477~1427 cm^−1^) followed by transformation to 2^nd^ derivative forms (lower graph). (**b**) The urine 1545 cm^−1^ peak was integrated by measuring the peak area between 1558 and 1532 cm^−1^.

**Figure 2:**
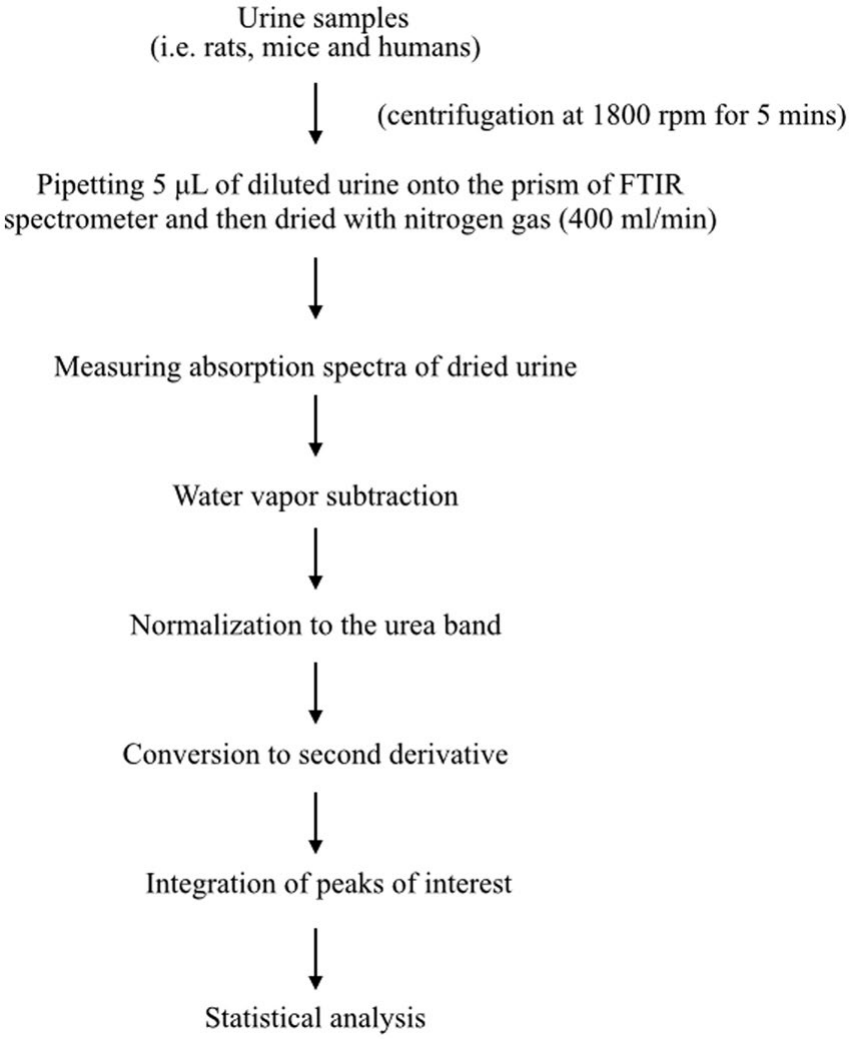
Protocol for urine handling and analysis. Urine is firstly centrifuged and the supernatant is diluted with distilled water and a 5 μl sample is dried on to the ATR prism surface. After drying, a sample spectrum is recorded. The raw sample spectra are pre-processed by water vapor subtraction, normalization to the peak of urea and second derivative conversion. Features of interest are then quantitated by integration and statistical assessment (Origin Pro 9.1).

**Figure 3:**
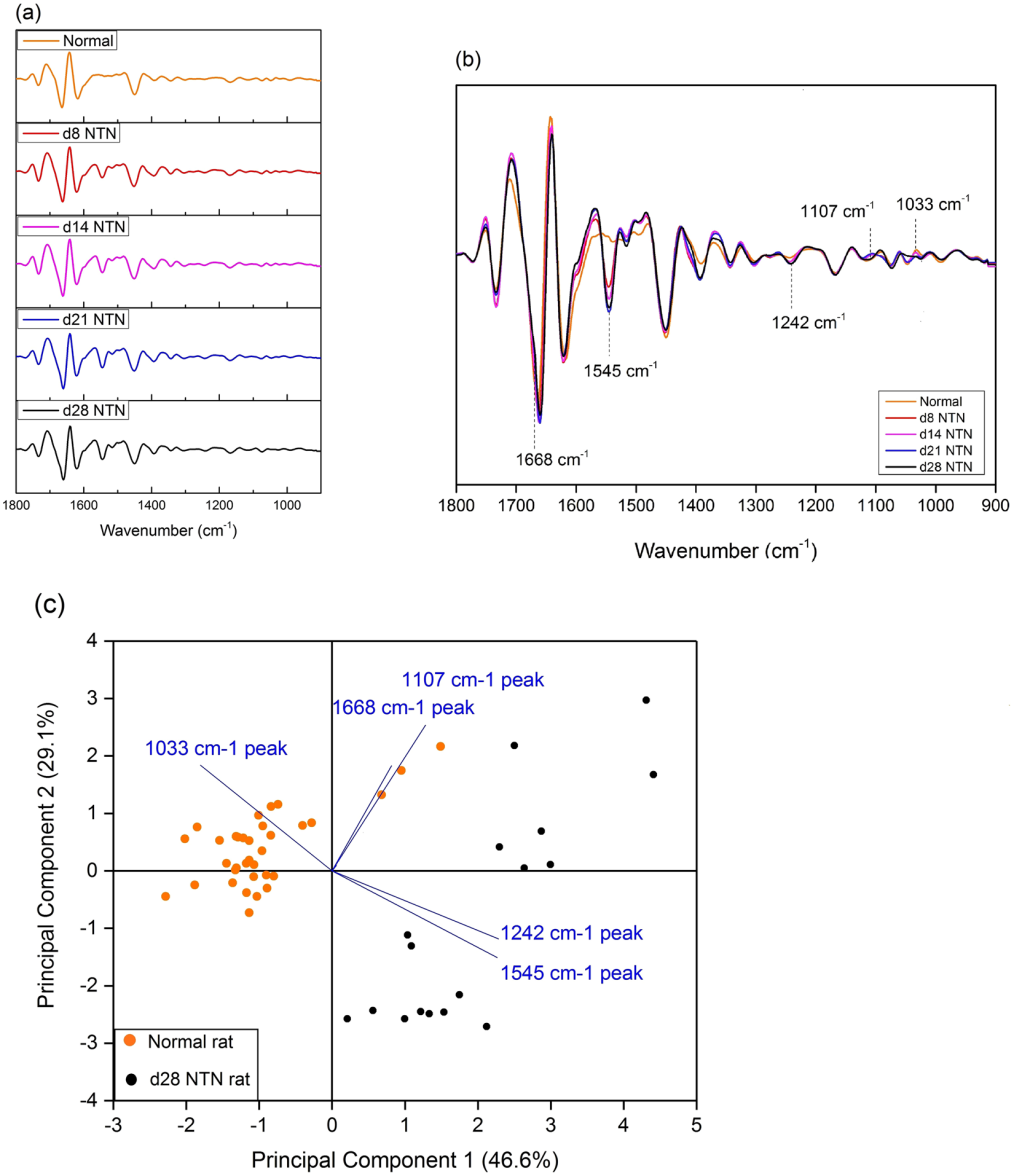
Five features of interest identified in experimental glomerulonephritis. (**a**) The average normalized second derivative FTIR spectra of urine obtained from normal WKY rats and different stages of NTN rats. (**b**) Peaks of interest (1668 cm^−1^, 1545 cm^−1^, 1242 cm^−1^, 1107 cm^−1^, and 1033 cm^−1^) potentially relevant to renal inflammation and/or fibrosis. (**c**) The PCA bioplot displays the correlations between the five spectral markers and individual urine sample of normal rats (n = 34) and d28 NTN rats (n = 18, there was one outlier excluded from this group). PC1 and PC2 explained 75.7% of total variance.

Absorbance spectra were pre-processed by removal of any contribution from water vapor, followed by normalization to the peak of urea at 1450 cm^−1^ and conversion to the second derivative forms Fig. 1(a). The rationale for choosing urea rather than creatinine for spectral normalization is that urea is a dominant component of urine FTIR spectra and is a reasonable measure of urine concentration, whereas in small animals such as rats and mice, the spectrum for urinary creatinine is too weak to provide a reliable measure of urinary dilution. Next, features of interest were quantified by integration between two frequency limits and intensities were compared between different disease states or treatment groups Fig. 1(b). The scheme of FTIR spectral acquisition and analysis of urine is illustrated in Fig. 2. Comparisons of the average normalized second derivative spectra of each nephritic group with that of the healthy control revealed six features of interest as possible markers to distinguish normal from nephritic groups Fig. 3(a) and (b). Integrated areas of these features were obtained from individual samples [i.e. normal rats (n = 34), day 8 NTN rats (n = 20), day 14 NTN rats (n = 18), day 21 NTN rats (n = 17) and day 28 NTN rats (n = 18)]. The results were then analyzed by a pairwise principal component analysis (PCA) for exploration of the most significant spectral responses in the multivariate IR data set, and discrimination of spectroscopic changes between different experimental groups. In addition, the one-way Analysis of Variance (ANOVA) followed by Fisher’s Least Difference Significant (LDS) *post hoc* test was conducted to verify the significance of the selected spectral markers (Table 1). The data are expressed as means with standard deviations (SD). The level of statistical significance considered *p* < 0.05. Origin Pro 9.1 was used for all the statistical analyses.

**Table 1:** Summary of potential IR marker bands, proteinuria, creatinine and urea concentrations in urine samples of normal and NTN rats.

Parameters	Frequency limits for integration		Normal rats (n = 34)	D8 NTN rats (n = 20)	D14 NTN rats (n = 18)	D21 NTN rats (n = 17)	D28 NTN rats (n = 18)
Urinary 1668 cm^−1^ peak	1676–1664 cm^−1^	Peak intensity	30.5 ± 1.6	10.5 ± 1.8*	12.6 ± 2.1*	27.1 ± 3.8	32.0 ± 4.0
		*p*-value (nephritis *vs*. normal)		<0.001	<0.001	0.342	0.670
Urinary 1545 cm^−1^ peak	1558–1532 cm^−1^	Peak intensity	17.0 ± 1.6	249.2 ± 13.5*	314.0 ± 13.4*	369.8 ± 17.3*	350.3 ± 24.2*
		*p*-value (nephritis *vs*. normal)		<0.001	<0.001	<0.001	<0.001
Urinary 1242 cm^−1^ peak	1254–1230 cm^−1^	Peak intensity	15.8 ± 0.7	30.9 ± 1.7*	31.8 ± 1.6*	37.0 ± 1.6*	33.9 ± 1.5*
		*p*-value (nephritis *vs*. normal)		<0.001	<0.001	<0.001	<0.001
Urinary 1107 cm^−1^ peak	1122–1092 cm^−1^	Peak intensity	26.3 ± 2.9	21.1 ± 3.4	21.3 ± 2.9	21.0 ± 2.7	30.7 ± 5.0
		*p*-value (nephritis *vs*. normal)		0.245	0.282	0.263	0.349
Urinary 1033 cm^−1^ peak	1047–1022 cm^−1^	Peak intensity	40.9 ± 1.9	33.4 ± 3.9*	36.1 ± 3.6	11.9 ± 1.6*	17.7 ± 3.1*
		*p*-value (nephritis *vs*. normal)		0.044	0.215	<0.001	<0.001
Proteinuria (mg/dl)		*p*-value (nephritis *vs*. normal)	3.7 ± 0.3	77.4 ± 6.9*	106.6 ± 4.2*	151.3 ± 9.1*	109 ± 9.8*
				<0.001	<0.001	<0.001	<0.001
Urine creatinine(mmol/L)		*p*-value (nephritis *vs*. normal)	4.7 ± 1.7	3.7 ± 1.1*	3.3 ± 1.3*	3.2 ± 0.7*	2.6 ± 0.3*
				0.008	<0.001	<0.001	<0.001
Urine urea (mmol/L)		*p*-value (nephritis *vs*. normal)	457.5 ± 146.8	304 ± 79.7*	256.1 ± 78.4*	236.9 ± 37.3*	234.1 ± 43.1*
				<0.001	<0.001	<0.001	<0.001

The data were expressed as mean ± SD and analyzed by one-way ANOVA followed by Fisher’s LSD *post hoc* test. Statistical significance (*) was set as p-valve < 0.05.

## Results

### Three potential spectral markers (i.e. the bands at the positions of 1668 cm^−1^, 1545 cm^−1^ and 1033 cm^−1^) were identified in dried urine that related to inflammation, progression of GN and chronic renal fibrosis, respectively

**Figure 4:**
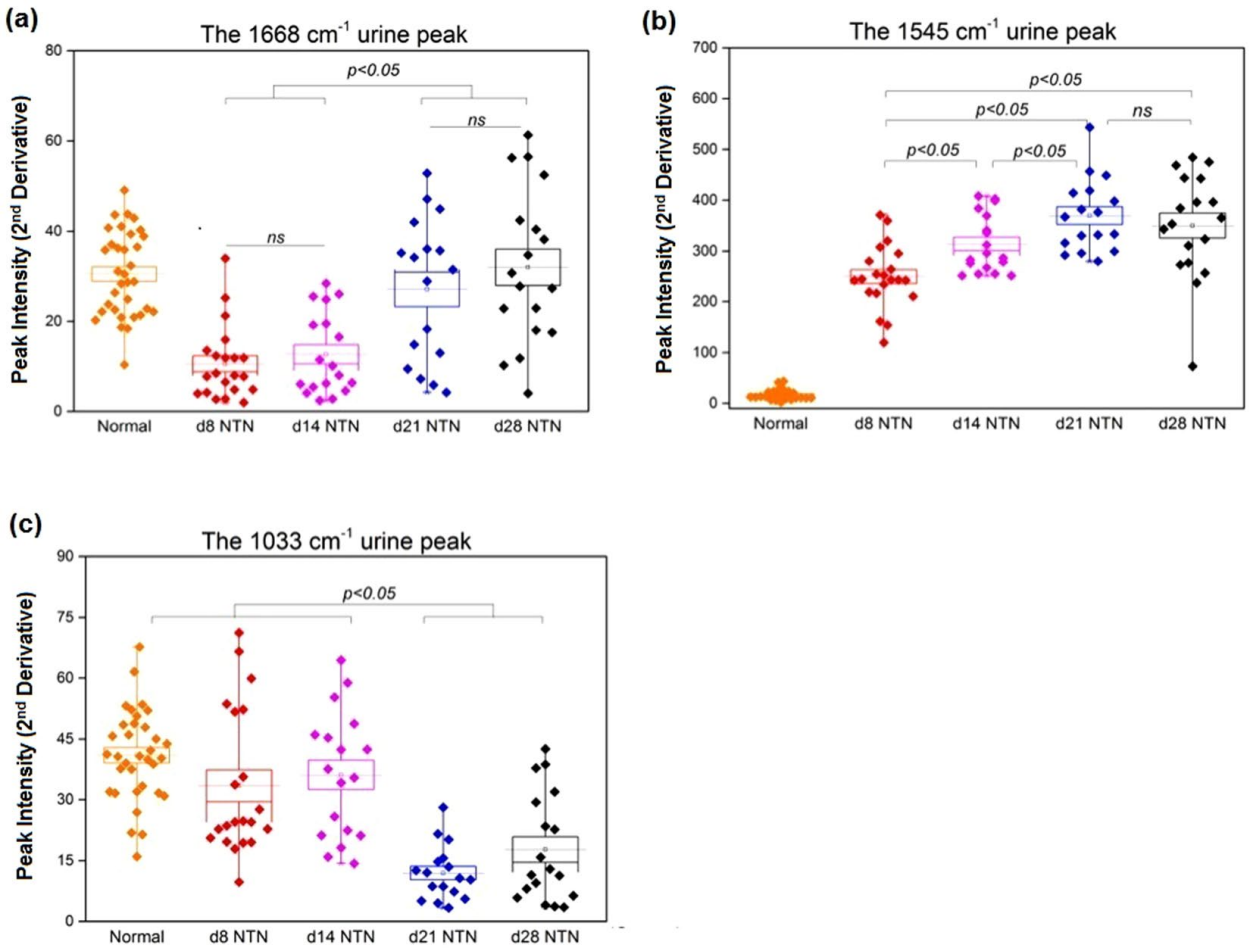
The 1668 cm^−1^, 1545 cm^−1^ and 1033 cm^−1^ IR peaks behavior in relation to renal inflammation, the progression of renal injury and renal fibrosis. The intensity of the urinary 1668 cm^−1^ spectral marker (**a**) was significant decreased in both d8NTN and d14 NTN groups (inflammation) compared with the normal group and the fibrotic group (i.e. d21NTN and d28NTN). The intensity of the urinary 1545 cm^−1^ spectral marker (**b**) increased as nephritis progressed. The 1033 cm^−1^ spectral marker (**c**) showed a marked reduction in the fibrotic group compared to the normal and inflammatory groups. (Boxes are mean ± SD. Data was analyzed by one-way *ANOVA* and subsequent Fisher’s LSD *post hoc* test for mean comparisons).

**Figure 5:**
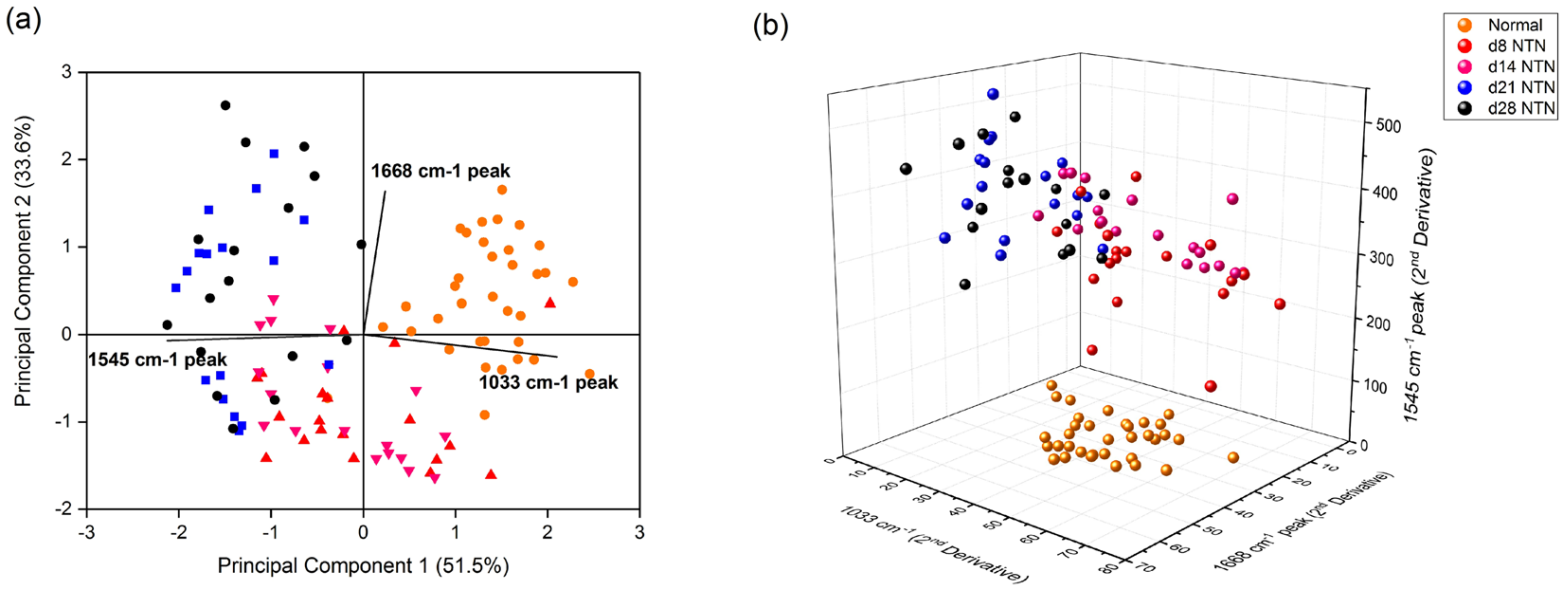
(**a**) The PCA biplot represents the locations of 2^nd^ derivative spectra of 107 urine samples and the correlations among the three potential urinary spectral markers (1668 cm^−1^, 1545 cm^−1^ and 1033 cm^−1^ bands) on the first two principal components (85.1% of total variance). **(b)** 3D scatter plot displayig the separation of normal, inflammatory and fibrotic NTN groups.

Initially five potential peaks of interest were identified by comparisons of the average normalized second derivative spectra of each nephritic group with that of the healthy control Fig. 3(a) and (b). Subsequently, PCA analysis was used for differentiation of these spectral data into two categories, normal rats *versus* rats at day 28 NTN. The PCA loadings and scatter plots displayed the five spectral features and individual samples on the first two components Fig. 3(c). The first principal component (PC1) (46.6%), the second principal component (PC2) (29.1%) and the third principal component (PC3) (14.9%) accounted for 90.6% of total variance for the six spectroscopic changes. The loading plot also illuminated that the spectral features at 1545 cm^−1^ and 1242 cm^−1^ were associated with PC1 while the spectral features a 1107 cm^−1^ and 1033 cm^−1^ were associated with PC2. The 1668 cm^−1^ spectral marker was more related to PC3. In Table 1, it showed the results of quantitation of these five features and the concentrations of urine urea and creatinine. The statistical significances of all five possible urinary spectral markers were further tested by one-way ANOVA and Fisher’s LDS *post hoc* test (Fig. 4), and three of the five IR features were found to be were statistically significant as potential biomarkers of kidney injury in experimental GN. In Fig. 5(a), the PCA biplot simultaneously displays information on locations of individual samples and correlations among these three potential IR features: the 1668 cm^−1^, 1545 cm^−1^ and 1033 cm^−1^ bands. PC1 (51.5% of total variance) appeared to correlate strongly with the 1545 cm^−1^ and 1033 cm^−1^ spectral markers, which had loadings with largest negative and positive magnitudes, respectively. PC2 (33.6% of total variance) had a higher correlation with the 1668 cm^−1^ spectral marker. This spectral pattern correlation was illustrated in a three-dimensional (3D) scatter plot in Fig. 5(b).

Taken together, the first candidate marker was the band at 1668 cm^−1^. Its band intensities in both day 8 and d14 NTN groups (inflammatory groups) were clearly distinguishable from those in the normal and fibrosis groups (i.e. day 21 and day 28 NTN rats) Fig. 4(a). The second candidate was the 1545 cm^−1^ band Fig. 4(b). Its peak intensity in each nephritic group (i.e. day 8, day 14, day 21 and day 28 NTN rats) was significantly higher than in normal healthy rats (*p* < 0.05). Moreover, its intensity continued to increase in accordance with the progression of GN injury, except for a slight reduction in the day 28 NTN group. The third peak of interest was the band at 1033 cm^−1^ Fig. 4(c). Its differences in intensity between normal, day 8 NTN and day 14 NTN groups were not significant. Nevertheless, there was a significant decrease in the peak intensity observed in both the day 21 NTN and day 28 NTN groups compared with either the normal group or the inflammatory groups (i.e. day 8 NTN and day 14 NTN rats) (*p* < 0.05).

These findings indicate that these spectral features 1668 cm^−1^, 1545 cm^−1^ and 1033 cm^−1^ may be useful urinary biomarkers of acute inflammatory renal injury and renal fibrosis.

### The intensities of the 1545 cm^−1^ and 1033 cm^−1^ spectral markers were altered in response to corticosteroid treatment in NTN rats

**Figure 6:**
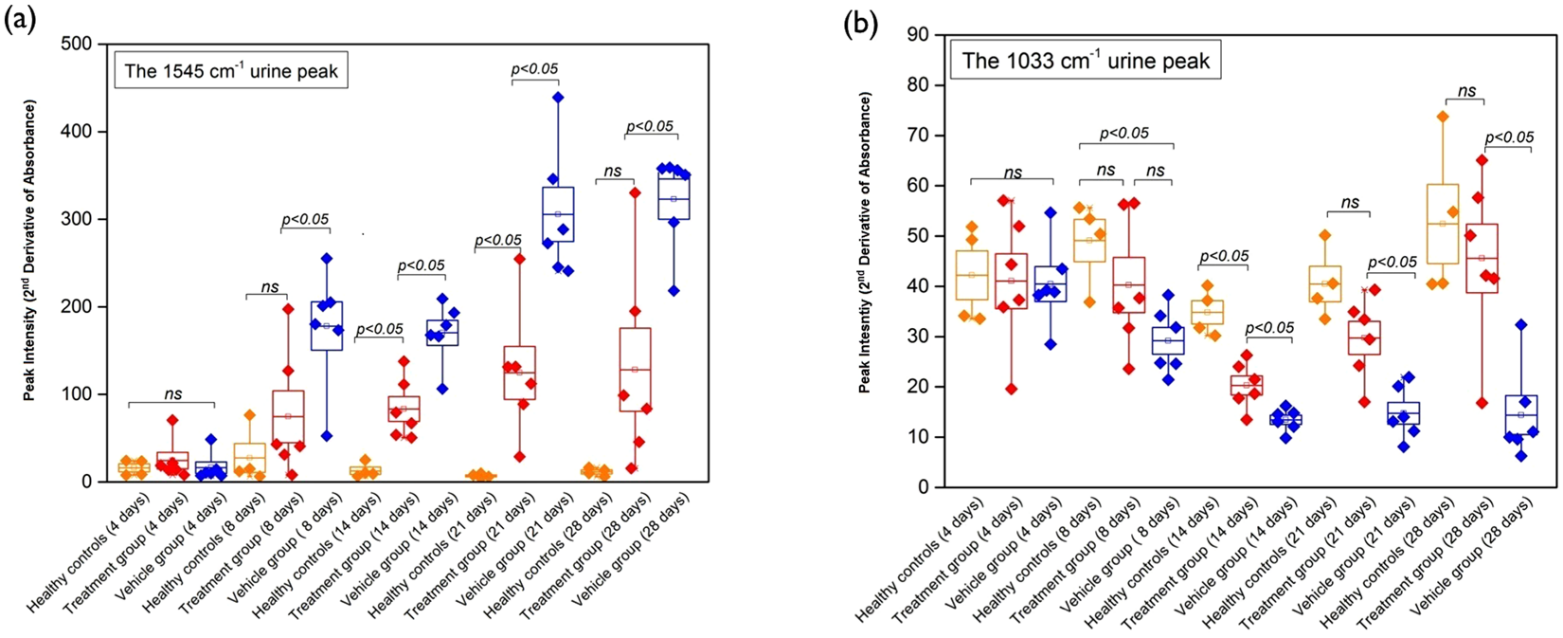
(**a**) Longitudinal study of the intensity of the urinary 1545 cm^−1^ band in NTN rats treated with DXM (started after urine collections on day 4) (red) compared with the normal (orange) and vehicle-treated NTN rats (blue). It was apparent that the peak intensities were reduced in response to DXM treatment. (**b**) Longitudinal analysis of the intensity of the urinary 1033 cm^−1^ band marker in the treatment group (red) compared with healthy controls (orange) and the vehicle group (blue). From day 14 to day 28, the vehicle treated group had a major reduction in the peak intensity whereas these changes in the spectral peaks were less in the treatment group (*p* < 0.05).

In the corticosteroid treatment experiments, the healthy control group had a constant intensity level of of the urinary 1545 cm^−1^ spectral marker, whereas in the vehicle treated nephritic (NTN) group, its intensity increased over time as GN injury progressed Fig. 4(b). Moreover, the intensity of the urinary 1545 cm^−1^ spectral marker declined significantly in response to dexamethasone (DXM) treatment in the NTN rats Fig. 6(a). The intensity of the urinary 1033 cm^−1^ spectral marker remained constant in the healthy control group, whereas in the vehicle treated NTN group its intensity declined over time Fig. 4(c). However, the peak intensity in the DXM treated group increased following administration of DXM Fig. 6(b). There was no consistent change in the urinary 1668 cm^−1^ spectral marker in the DXM treated NTN group. These findings suggest that FTIR can be used to monitor a therapeutic response in inflammatory GN by detecting changes in the 1545 cm^−1^ and/or 1033 cm^−1^ spectral biomarkers in urine.

### The urinary 1545 cm^−1^ spectral marker had a positive correlation with proteinuria during the time course of NTN and was more reliable in assessing the severity of renal injury

**Figure 7:**
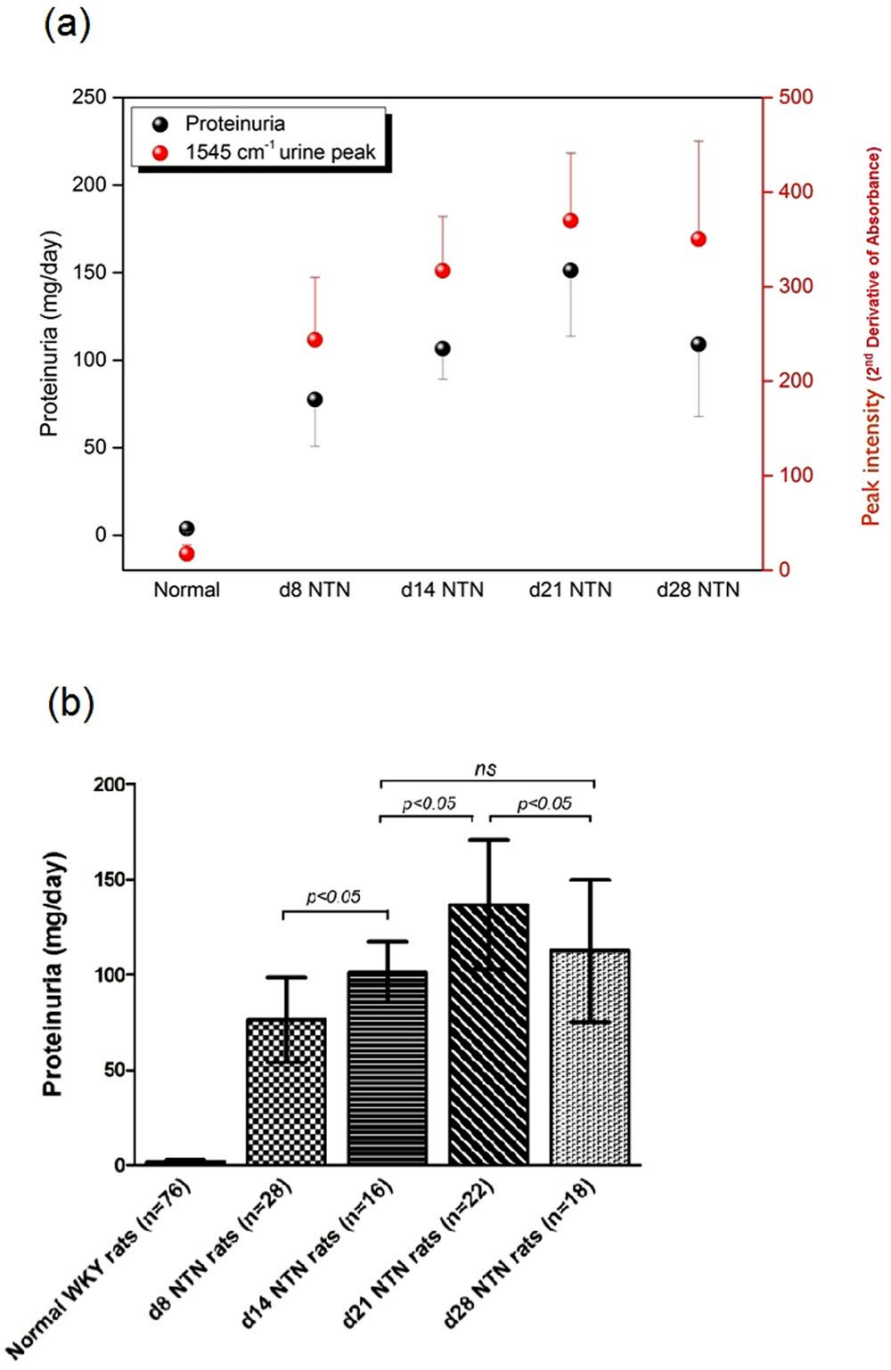
(**a**) The urinary 1545 cm^−1^ mid-infrared marker had a positive correlation with proteinuria during the time course of NTN. (**b**) Although the level of proteinuria increased gradually along with progression of nephritis, there was a manifest sudden drop at d28 NTN (*p* < 0.05) probably caused by a fall in glomerular filtration rate due to severe renal injury.

Figure 7(a) compares the longitudinal patterns of the urinary 1545 cm^−1^ spectral marker with the development of proteinuria in the NTN model. During earlier stages of renal injury its peak intensity correlated with the degree of proteinuria as NTN injury progressed. Figure 7(b) shows that the level of proteinuria increased gradually as NTN evolved, but that there was a significant drop at day 28 of NTN (p < 0.05) when renal failure has become established in this model.

### The urinary 1545 cm^−1^ spectral marker was also useful in detecting severe murine lupus nephritis

**Figure 8:**
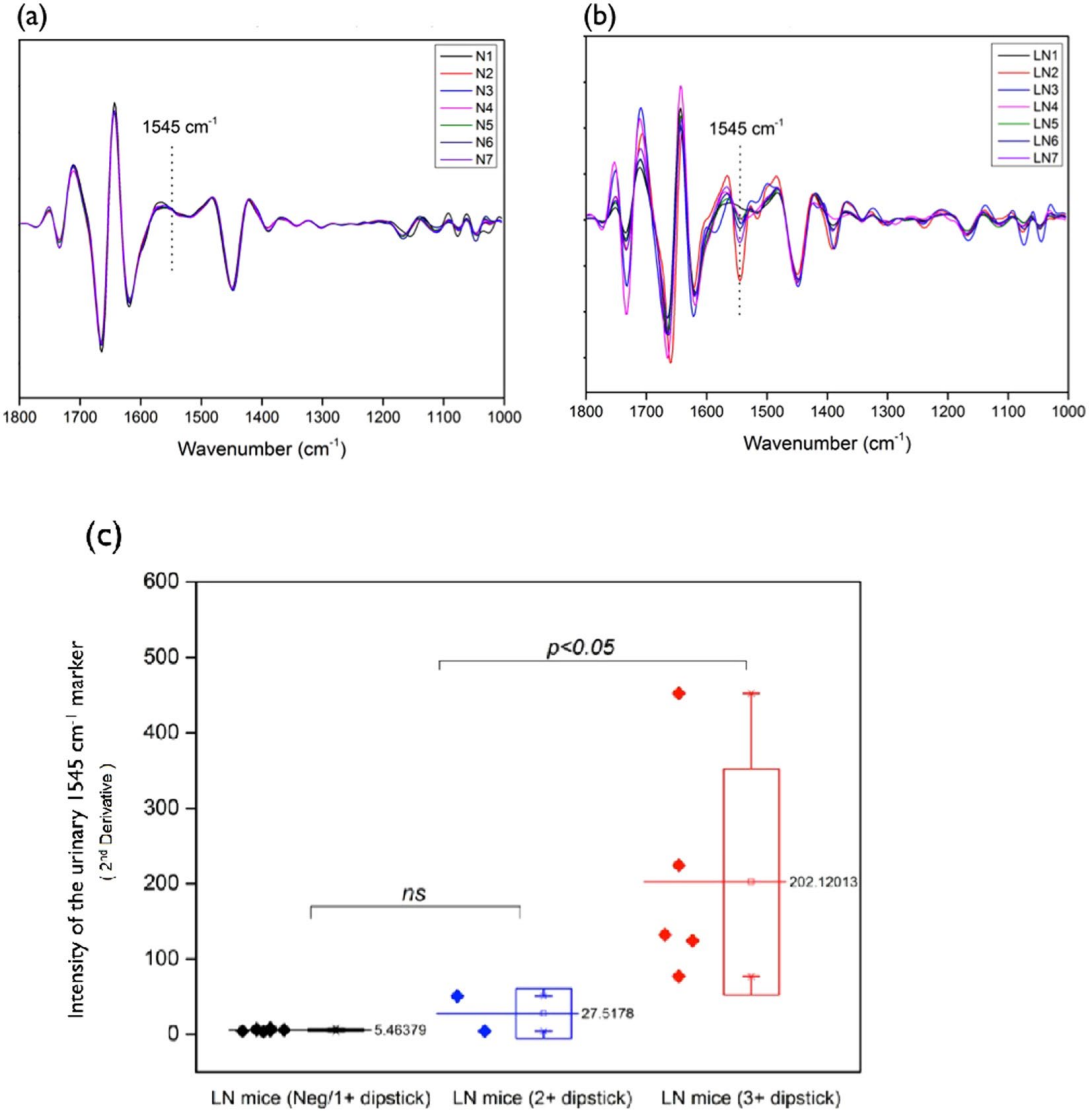
(**a**) Normalized 2^nd^ derivative IR spectra of seven (NZB x NZW) F1 mice with negative to 1 + protein by dipsticks (i.e. negative to trace protein for the samples including N1, N2, N4, N5, N6 and N7, and 1 + proteinuria for the sample N3 (**b**) Normalized 2nd derivative IR spectra of seven (NZB x NZW) F1 mice with 2 + proteinuria (the samples of LN1 and LN4) and 3 + proteinuria (the samples of LN2, LN3, LN5, LN6 and LN7). (**c**) The intensity of the urinary 1545 cm^−1^ spectral marker in LN mice with negative/1 + protein (n = 7), LN mice with 2 + protein (n = 2) and LN mice with 3 + protein (n = 5). The mice with significant proteinuria (3 + positive) had the highest level of the 1545 cm^−1^ peak intensity compared to the other two groups, but there was no significant difference between mice with negative and mild proteinuria (≤2 + protein). (The data are expressed as mean ± SD, analyzed by one way *ANOVA* with Fisher’s LSD *post hoc* test)

(NZB x NZW) F1 mice develop spontaneous autoimmune GN with varying severity. Therefore, we investigated whether the urinary FTIR urine spectral biomarkers could be used to assess the severity of murine lupus nephritis. The FTIR spectra of urine obtained from 14 (NZB x NZW) F1 mice were analyzed following the method described above and for the same potential spectral marker bands. Figure 8(a) and (b) illustrate the normalized 2^nd^ derivative spectra of 7 mice with negative to 1 + proteinuria on dipstick and with 2 + to 3+/4+ proteinuria on dipstick, respectively. The 1545 cm^−1^ spectral marker was found to correlate with the extent of lupus nephritis according to the degree of proteinuria, as shown in Fig. 8(c). (NZB x NZW) F1 lupus mice with 3 + /4 + proteinuria had a statistically higher peak intensity than either the mice without proteinuria or with only mild proteinuria (the range from 1 + to 2 + positive in urine dipstick); the peak intensity difference between lupus mice with negative proteinuria and with mild proteinuria was not significant. These findings indicate the potential for the urinary 1545 cm^−1^ spectral marker to be a diagnostic biomarker of severe lupus nephritis.

### The intensity of the urinary 1545 cm^−1^ spectral marker was also significantly higher in patients with moderate/severe crescentic GN

**Figure 9:**
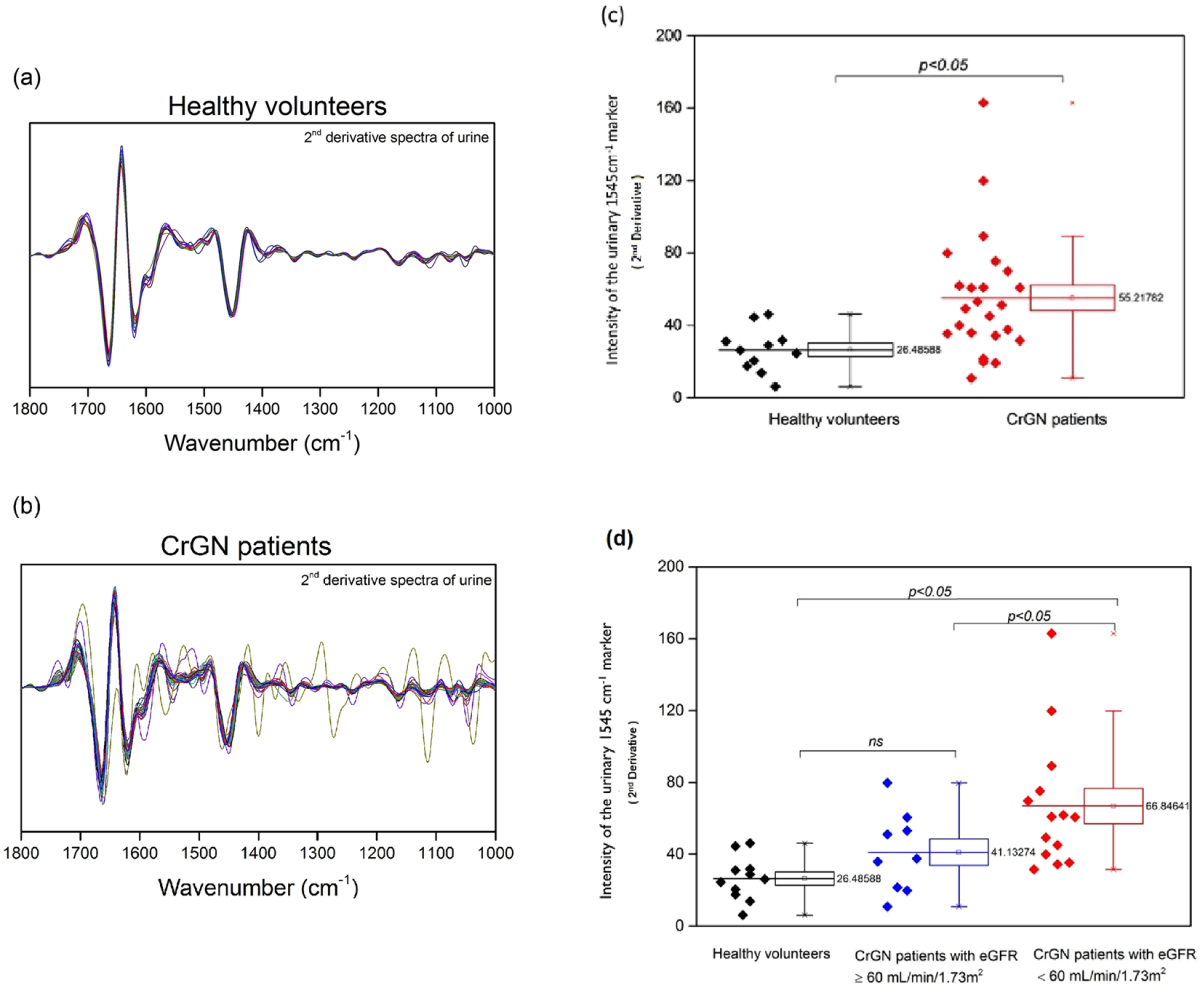
Comparison of FTIR urine spectra of patients with crescentic glomerulonephritis (CrGN) with those from healthy volunteers. The normalized 2^nd^ derivative spectra of urine of (**a**) healthy volunteers (n = 11) and (**b**) CrGN patients (n = 24) are shown. The 1545 cm^−1^ band was significantly higher in CrGN patients [*p* < 0.05, graph (**c**)]. (**d**) The intensity of the 1545 cm^−1^ band is significant higher in patients with severe/moderate CrGN patients (eGFR < 60 ml/min/1.73 m^2^) than in both healthy volunteers and mild CrGN patients (eGFR ≥ 60 ml/min/1.73 m^2^). (The data are expressed as mean ± SD as determined by sample *t* test or one-way *ANOVA* with Fisher’s LSD *post hoc* test).

The intensity of the urinary 1545 cm^−1^ spectral marker was consistently different between healthy volunteers and 24 patients with crescentic GN who were diagnosed with ANCA associated vasculitis Fig. 9(a) and (b). Overall, its intensity was elevated in the GN patients compared with healthy volunteers (*p* < 0.05) Fig. 9(c). As shown in Fig. 9(d), it was also evident that moderate-to-severe GN patients with an eGFR were <60 ml/min/1.73 m^2^ and active disease or in remission had a higher 1545 cm^−1^ band intensity than GN patients with eGFR ≥ 60 ml/min/1.73 m^2^.

### The 1545 cm^−1^ band of the urine ultrafiltrates collected from AMICON Ultra centrifugal filters of MWCO 100 K increased its intensity with the severity of NTN

**Figure 10:**
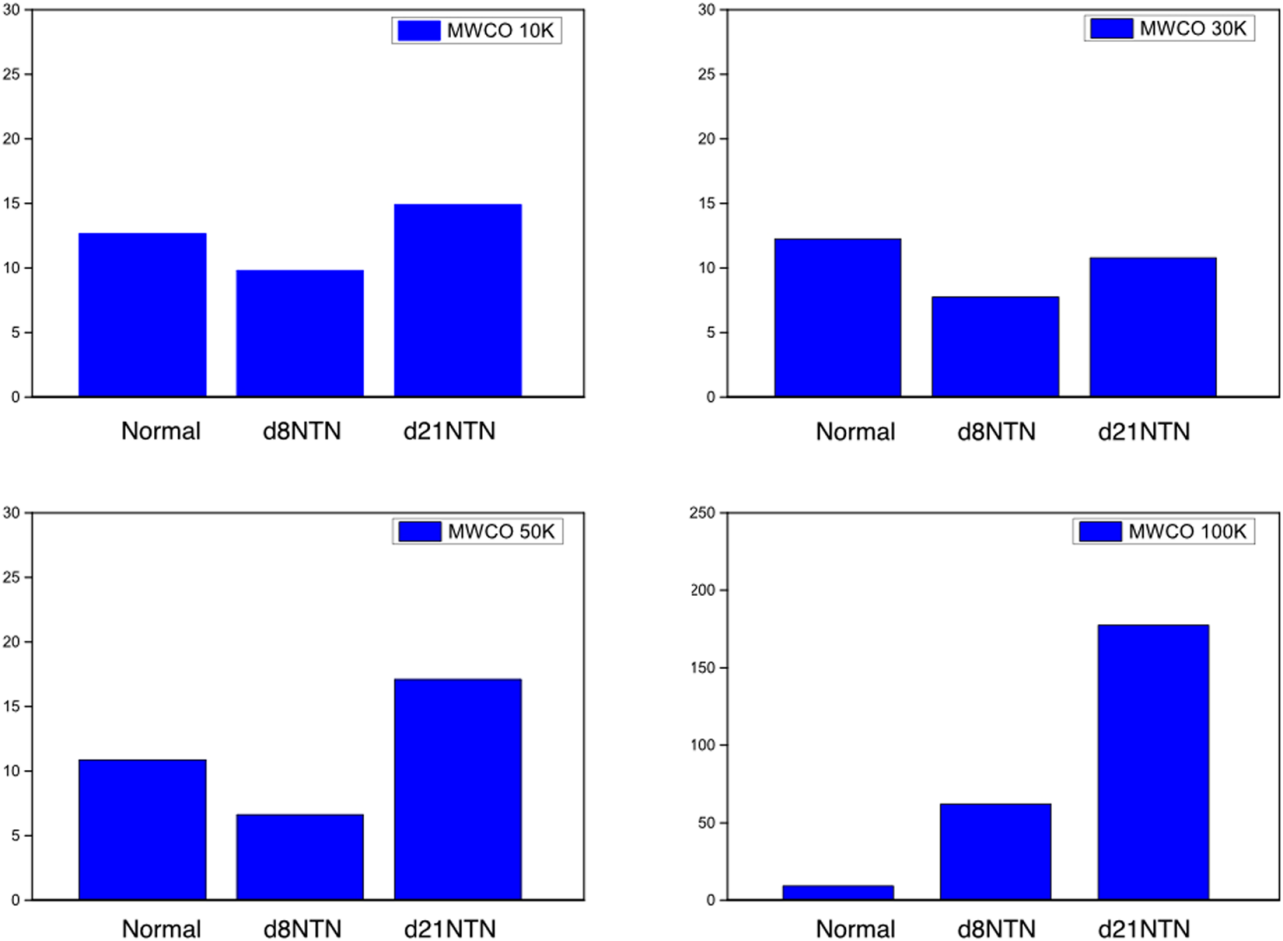
The 1545 cm^−1^ peak intensity of the urine ultrafiltrates. Urine samples of normal, d8 NTN and d21 NTN rats were filtered through AMICON centrifugal filters with different M cutoffs of 10 K, 30 K, 50 K and 100 K, and the ultrafiltrates were analyzed. The 1545 cm^−1^ component was only observed in the ultrafiltrate collected from MWCO 100 AMICON filter as it increased gradually from normal, d8 NTN (inflammation) to d21 NTN (fibrosis).

Figure 10 shows that the 1545 cm^−1^ band in the urine ultrafiltrates collected from 100 K AMICON centrifugal filters increases intensity from normal, day 8 NTN to day 21 in NTN rats, consistent with the results during NTN progression of Fig. 4(b). The nephritic urine ultrafiltrates obtained from 10 k, 30 k and 50 K AMICON centrifugation devices did not show an increase of the 1545 cm^−1^ band.

## Discussion

By applying ATR-FTIR spectroscopy to urine samples from rodent models of acute inflammatory GN, we have identified several characteristic urinary spectral markers of acute and chronic renal injury. Of these, the urinary 1545 cm^−1^ band was also diagnostically applicable to murine lupus nephritis and human GN. The same biomarkers were also useful for monitoring the therapeutic response to steroid therapy.

Several studies have reported the application of FTIR to routine clinical chemistry analyses and disease diagnostics in fluids, cells and tissues^24, 29–33^. Along with existing applications in various types of cancer, bone and neurodegenerative diseases^34–36^, there are ongoing initiatives to encourage work on infrared based technologies and their wider application to clinical medicine^37^ (https://clirspec.org/uk-network/). However, so far there are few applications to kidney diseases. Two recent reports have described renal tissue FTIR using renal biopsy material to detect and quantify fibrosis and inflammation, and to diagnose diabetic nephropathy, but in neither report were there corresponding or matching analyses of urine^20, 21^. Application of FTIR spectroscopy to tissue analyses is a potentially useful adjunct to current histological techniques for refining pathological classification of renal disease, but it still depends on obtaining renal tissue samples invasively. By contrast, our study has focused on detecting potentially easy to acquire urinary biomarkers of renal injury in a reproducible rodent model of inflammatory and progressive GN, as well as their validation in a cohort of patients with crescentic GN.

The band at 1545 cm^−1^ is likely to be attributable to the amide II band of the peptide bonds of urinary protein. Hence, it is a measure for urinary protein and has the potential to be an earlier and more sensitive marker of progression of renal injury than currently measured proteinuria. In the model of NTN in WKY rats, albuminuria can be detected by rocket immunoelectrophresis on day 4 following injection of nephrotoxic serum^27^. The Amicon ultracentrifugal experiments reported here indicate that the protein responsible was only observed in the urine ultrafiltrate collected from 100 K AMICON Ultra filter (Fig. 10). This implies that its relative molecular mass is >50 kDa, which could be albumin (M_r_ = 68 kDa)^38^ or other high M_r_ protein. In relation to the bands at 1668 cm^−1^ and 1033 cm^−1^, the majority of the absorption around 1668 cm^−1^ band probably comes from urea and creatinine. There will also be amide I band of protein present, but this will be no greater than the amide II in intensity. If the change in 1668 cm^−1^ band is due to protein, then its changes should match changes in the 1545 cm^−1^ amide II band. The 1033 cm^−1^ band could be carbohydrate, ribose of RNA, phosphate free or in lipid and other possibilities. However, the FTIR spectra of biological systems are very complex due to the overlapping absorption of multiple components. Based on contemporarily available data in IR libraries, the information seems insufficient to elucidate the molecular models corresponding to these potential urinary spectral markers and further work is needed.

The standardized method of analyzing FTIR spectra of dried urine samples described here is applicable and optimal for both animal (e.g. rats and mice) and human samples. Three characteristic urinary spectral makers (1668 cm^−1^, 1545 cm^−1^ and 1033 cm^−1^) were identified in experimental GN, which seem to be linked to the acute inflammatory, progressive and fibrotic phases of this GN model, respectively. As to potential for translation to the clinic, the 1545 cm^−1^ peak is the most clear and universal marker of progressive kidney diseases. This is because this spectral marker was evident in the rat and mouse models of crescentic GN and lupus nephritis, as well as in patients with ANCA-associated vasculitis and GN. A larger number of patients with more diverse causes of kidney disease will be need to be explored to establish the value of the spectral markers we have identified in the present study, and to test the wider application of FTIR technique in clinical nephrology. However, the relative simplicity and convenience in handling urine samples, and the potential for a higher throughput, compared with other spectroscopic methods currently being applied to urine, makes this a worthwhile endeavor.

## References

[1] Wetmore JB, Collins AJ (2016). Global challenges posed by the growth of end-stage renal disease. Renal Replacement Therapy.

[2] Inker LA (2016). GFR estimation using β-trace protein and β_2_-microglobulin in CKD. Am. J. Kidney Dis..

[3] Doi K (2009). Reduced production of creatinine limits its use as marker of kidney injury in sepsis. J. Am. Soc. Nephrol..

[4] Levey AS (2009). Proteinuria as a surrogate outcome in CKD: report of a scientific workshop sponsored by the national kidney foundation and the US food and drug administration. Am. J. Kidney. Dis..

[5] Miller WG (2009). Current issues in measurement and reporting of urinary albumin excretion. Clin. Chem..

[6] Smith, B. C. Fundamentals of Fourier transform infrared spectroscopy, second edition (CRC Press, 2011).

[7] Baker MJ (2016). Developing and understanding biofluid vibrational spectroscopy: a critical review. Chem. Soc. Rev..

[8] Baker MJ (2014). Using Fourier transform IR spectroscopy to analyze biological materials. Nat. Protoc..

[9] Severcan, F. & Haris, P. I. Vibrational spectroscopy in diagnosis and screening (IOS Press, 2012).

[10] Untereiner V (2014). Bile analysis using high-throughout FTIR spectroscopy for the diagnosis of malignant biliary stricture: a pilot study in 57 patients. J. Biophotonics..

[11] Gajjar K (2013). Fourier-transform infrared spectroscopy coupled with a classification machine for the analysis of blood plasma or serum: a novel diagnostic approach for ovarian cancer. Analyst..

[12] Hands JR (2016). Brain tumor differentiation: rapid stratified serum diagnostics via attenuated total reflection Fourier-transform infrared spectroscopy. J. Neurooncol..

[13] Travo A (2010). IR spectral imaging of secreted mucus: a promising new tool for the histopathological recognition of human colonic adenocarcinomas. Histopathology..

[14] Maziak DE (2007). Fourier-transform infrared spectroscopic study of characteristic molecular structure in cancer cells of esophagus: an exploratory study. Cancer Detect. Prev..

[15] Nallala J (2014). Infrared spectral histopathology for cancer diagnosis: a novel approach for automated pattern recognition of colon adenocarcinoma. Analyst..

[16] Gazi E (2003). Applications of Fourier transform infrared microspectroscopy in studies of benign prostate and prostate cancer. A pilot study. J. Pathol..

[17] Lewis PD (2010). Evaluation of FTIR spectroscopy as a diagnostic tool for lung cancer using sputum. BMC cancer.

[18] Tian P (2015). Intraoperative diagnosis of benign and malignant breast tissues by Fourier transform infrared spectroscopy and support vector machine classification. Int. J. Clin. Exp. Med..

[19] Hughes C, Brown MD, Clarke NW, Flower KR, Gardner P (2012). Investigating cellular responses to novel chemotherapeutics in renal cell carcinoma using SR-FTIR spectroscopy. Analyst..

[20] Vuiblet V (2016). Renal graft fibrosis and inflammation quantification by an automated Fourier-transform infrared imaging technique. J. Am. Soc. Nephrol..

[21] Varma VK, Kajdacsy-Balla A, Akkina SK, Setty S, Walsh MJ (2016). A label-free approach by infrared spectroscopic imaging for interrogating the biochemistry of diabetic nephroathy progression. Kidney Int..

[22] Guerra-López JR, Gűida JA, Della Védova CO (2010). Infrared and Raman studies on renal stones: the use of second derivative infrared spectra. Urol. Res..

[23] Schröder UC (2015). Rapid, culture-independent, optical diagnostics of centrifugally captured bacteria from urine samples. Biomicrofluidics..

[24] Mitchell AL, Gajjar KB, Theophilou G, Martin FL, Martin-Hirsch PL (2014). Vibrational spectroscopy of biofluids for disease screening or diagnosis: translation from the laboratory to a clinical setting. J. Biophotonics..

[25] Trevisan J, Angelov PP, Carmichael PL, Scott AD, Martin FL (2012). Extracting biological information with computational analysis of Fourier-transform infrared (FTIR) biospectroscopy datasets: current practices to future perspectives. Analyst..

[26] Kazarian SG, Chan KL (2013). ATR-FTIR spectroscopic imaging: recent advances and applications to biological systems. Analyst..

[27] Tam FW (1999). Development of scarring and renal failure in a rat model of crescentic glomerulonephritis. Nephrol. Dial. Transplant..

[28] Morel L, Wakeland EK (1998). Susceptibility to lupus nephritis in the NZB/W model system. Curr. Opin. Immunol..

[29] Oliver KV (2016). Infrared vibrational spectroscopy: a rapid and novel diagnostic and monitoring tool for cystinuria. Sci. Rep..

[30] Petibois C (1999). Determination of glucose in dried serum samples by Fourier-transform infrared spectroscopy. Clin. Chem..

[31] Sitole L, Steffens F, Krüger TP, Meyer D (2014). Mid-ATR-FTIR spectroscopic profiling of HIV/AIDS sera for novel systems diagnostics in global health. OMICS..

[32] Lee CH (2016). Arsenite regulates prolongation of glycan residues of membrane glycoprotein: A pivotal study via wax physisorption kinetics and FTIR imaging. Int. J. Mol. Sci..

[33] Miller LM, Dumas P (2010). From structure to cellular mechanism with infrared microspectroscopy. Curr. Opin. Struct. Biol..

[34] Schmidt FN (2017). Assessment of collagen quality associated with non-enzymatic cross-links in human bone using Fourier-transform infrared imaging. Bone..

[35] Paschalis EP, Gamsjaeger S, Klaushofer K (2017). Vibrational spectroscopic techniques to assess bone quality. Osteoporos. Int..

[36] Mordechai S, Shufan E, Porat Katz BS, Salman A (2017). Early diagnosis of Alzheimer’s disease using infrared spectroscopy of isolated blood samples followed by multivariate analysis. Analyst..

[37] Baker MJ, Faulds K (2016). Fundamental developments in clinical infrared and Raman spectroscopy. Chem. Soc. Rev..

[38] Lozano-Ramos I (2015). Size-exclusion chromatography-based enrichment of extracellular vesicles from urine samples. J. Extracell. Vesicles..

